# Effect of robotic tilt table verticalization on recovery in patients with disorders of consciousness: a randomized controlled trial

**DOI:** 10.1007/s00415-022-11508-x

**Published:** 2022-12-19

**Authors:** M. J. Rosenfelder, V. C. Helmschrott, L. Willacker, B. Einhäupl, T. M. Raiser, A. Bender

**Affiliations:** 1 Therapiezentrum Burgau, Kapuzinerstraße 34, 89331 Burgau, Germany; 2 Clinical and Biological Psychology, Institute of Psychology and Education, Ulm University, Albert-Einstein-Allee 47, 89069 Ulm, Germany; 3 Department of Neurology, Ludwig-Maximilians-University of Munich, Marchioninistraße 15, 81377 Munich, Germany

**Keywords:** Disorders of consciousness, ERIGO®, Tilt table, Verticalization, Functional recovery

## Abstract

Verticalization is a common therapeutic intervention during rehabilitation of patients with disorders of consciousness (DoC). The Erigo®Pro is a robotic tilt-table (RTT) with built-in stepping unit for the lower extremities to prevent orthostatic hypotension during verticalization. In addition, the system also provides functional electrical stimulation (FES) of muscles of the lower extremities. In this randomized controlled clinical trial (RCT), 47 patients with subacute DoC received a 4-week verticalization regime (16 verticalization sessions) and were allocated to one of three experimental groups: (1) verticalization by means of RTT with FES, (2) by means of RTT without FES, or (3) by conventional physiotherapy (CPT). Level of consciousness (LoC), spasticity, functional independence in daily activities, and functional brain connectivity measured by means of high-density quantitative EEG were assessed at baseline, directly after the verticalization program and after 6 months. There was a similar clinical improvement in all three experimental groups. RTT was not associated with an effect on any of the clinical outcomes. Verticalization or mobilization time during the study period was significantly positively correlated with recovery of consciousness (rho = 0.494, *p* < 0.001) in the short term and showed a statistical trend at the 6 months follow-up (rho = 0.244, *p* = 0.078). In conclusion, RTT treatment is not more effective in promoting recovery of consciousness than CPT in subacute DoC patients. Yet, our data suggest, that verticalization may be an important and feasible rehabilitation intervention in this group of patients. ClinicalTrials.gov NCT Number NCT02639481, registered on December 24, 2015.

## Introduction

Patients with brain injuries resulting from stroke, hypoxic ischemic encephalopathy (HIE), or traumatic brain injury (TBI) often suffer from prolonged DoC in the form of the unresponsive wakefulness syndrome (UWS) or the minimally conscious state (MCS) [1, 2].

So far, only few therapies have shown limited promise to improve the state of consciousness and the clinical outcome in this group of patients [3]. Therefore, the clinical need for evidence-based therapies to improve DoC outcome is substantial. Bringing DoC patients into an upright, verticalized body position has long been a method advocated by experienced therapists to further recovery of consciousness [4]. This treatment strategy is mainly based on experience rather than on scientific evidence, though. Verticalization is a safe and well-tolerated method [5], which can improve orthostatic tolerance [6, 7], cognitive function, global motor function, sensory motor, and vestibular system plasticity, e.g. in stroke patients [8]. Yet, verticalization bears the risk of causing synkopes if applied without simultaneous stepping function for the lower limbs [9].

A randomized-controlled trial demonstrated feasibility of verticalization training using a RTT device with integrated stepping function of the lower extremities, the Erigo®Pro, as part of a neurorehabilitation program for DoC patients [10]. Patients with severe acquired brain injuries profit from both conventional and verticalization treatment in a similar way, yet improvement associated with Erigo®Pro treatment appeared faster [11]. On an electrophysiological level, verticalization with passive stepping in MCS patients led to a significant post-treatment increase in beta-power of the EEG signal, which the authors related to elevated alertness through verticalization [12].

Moreover, in a systematic review on verticalization treatment in DoC, Erigo®Pro was found to reduce the orthostatic hypotension in chronic DoC patients [13].

The aim of this randomized controlled trial was to test the effect of 4 weeks of Erigo® Pro RTT treatment either with or without FES on neurologic outcome of subacute DoC patients in an inpatient neurorehabilitation setting compared to CPT.

## Methods

### Study design

We conducted a three-arms randomized controlled trial of 47 subacute DoC patients in the inpatient early neurorehabilitation setting of a rehabilitation hospital in the southern German state of Bavaria. After assessment of inclusion and exclusion criteria (s. below), patients were randomly assigned to one of the following three groups: (1) RTT with vertical tilt > 60°, with repetitive cyclic leg movements and with FES (RTT+F; *n* = 14); (2) RTT with vertical tilt > 60°, with repetitive cyclic leg movements and without FES (RTT-F; *n* = 15); and (3) conventional physiotherapy (CPT; *n* = 18).

The local ethics committee of the Ludwig-Maximilians-University of Munich approved the study and written informed consent was given by the patients’ legal guardians in accordance with the 1964 declaration of Helsinki. The study has been prospectively registered on ClinicalTrials.gov (NCT Number NCT02639481).

### Patients

Inclusion criteria were age 18–80 years, written informed consent of the legal guardian, an acquired brain injury, and a clinical state of UWS or MCS according to repeated Coma Recovery Scale-Revised (CRS-R) assessment [14]. All patients were in a DoC for at least 4 weeks post-injury.

Exclusion criteria were pre-existing DoC, continuous intravenous sedation with propofol or midazolam, body weight above 135 kg (due to technical constraints of the RTT device), length of the legs below 75 cm or above 100 cm (due to technical constraints), fixed contractures of lower joints (hip, knee, foot), instabilities in the bones (fractures, instability in the spine, osteoporosis, arthritis), lesions of the skin on lower extremities, and cardiological contraindications. Further, aggressive, and uncooperative behavior of the patient led to exclusion as well as disproportional growth of lower extremities and/or spine, vascular disease of lower extremities, pacemaker, or pregnancy.

### Intervention

Therapeutic intervention was performed over a period of four weeks. A final follow-up study visit was conducted at 6 months after inclusion. Four therapy sessions were performed per week and each therapy session took 45 min for all study groups. This led to a total therapy time of at least 12 h in four weeks (45 min × 4 days × 4 weeks).

**Fig. 1:**
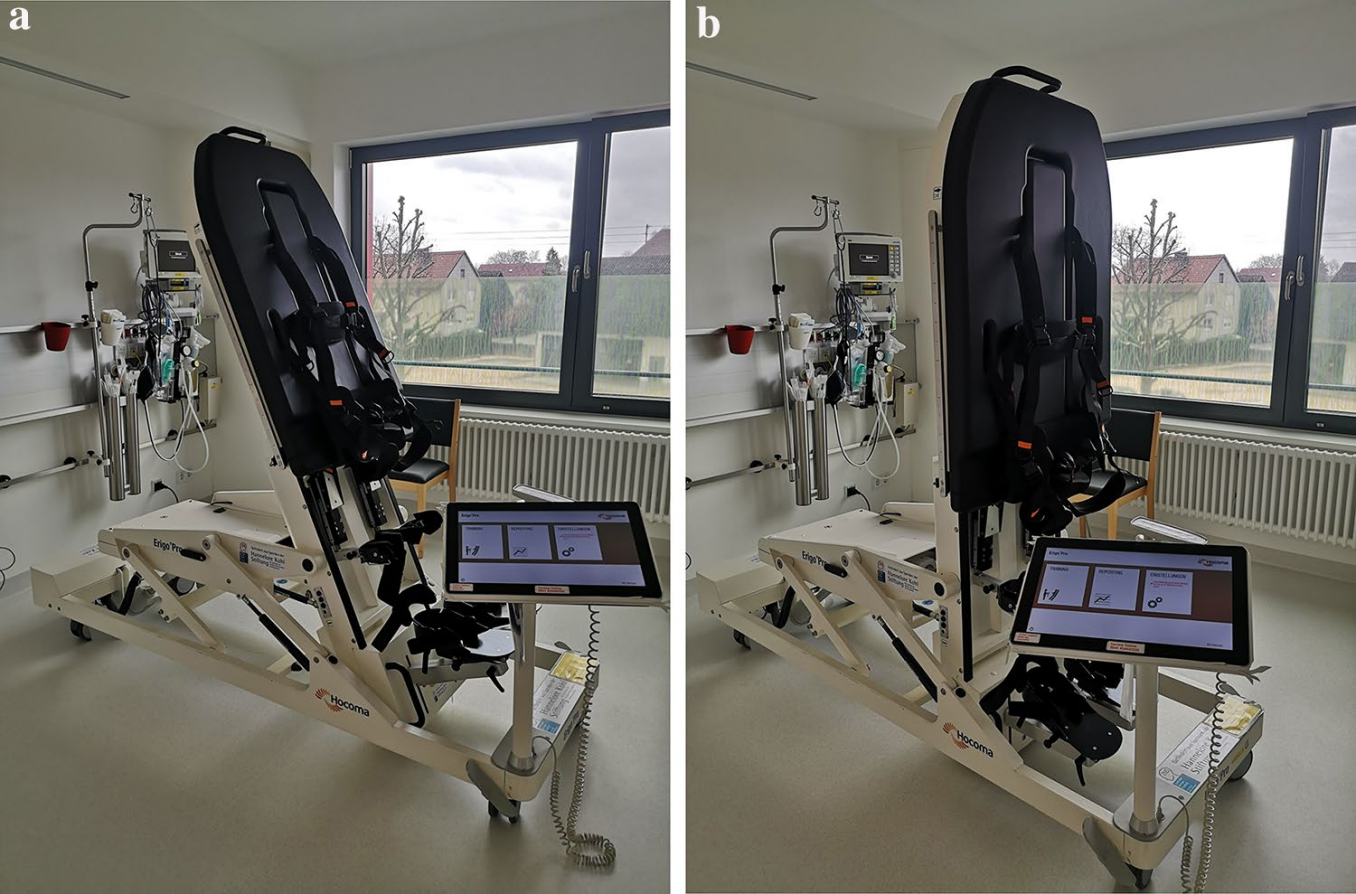
Erigo®Pro (Hocoma AG, Volketswil, Switzerland) robotic tilt table system with cyclic leg movement function with a) 75° and b) 90° tilt, respectively

In the two intervention groups (RTT + F, RTT-F), the ErigoPro® system (Hocoma AG, Volketswil, Switzerland, Fig. 1) was used for verticalization with robotic cyclic leg movements (Fig. 1). In both RTT groups, treatment parameters, such as range of motion (ROM; normal 45°) and cadence (min. 24 steps per minute, SPM), were adjusted for the Erigo®Pro according to the manufacturer's recommendations. Verticalization was set to minimum 60°; depending on cardiopulmonary parameters (respiratory rate, heart rate, blood pressure, oxygen saturation), vertical position was gradually increased (in 5° steps) to a maximum of 90°, as long as the above-named cardiopulmonary parameters of the patient remained stable. All stimulation parameters complied with the approval of the Erigo®Pro as a medical device with the corresponding CE marking.

In the RTT+F group, FES was performed with eight channels: M. quadriceps femoris (knee extension; large rectangle electrodes), M. biceps femoris (knee flexion; large rectangle electrodes), M. tibialis anterior (foot elevation; small oval electrodes), M. gastrocnemius (foot flexion; large rectangle electrodes) on both sides. The stimulation parameters of the FES were selected for all electrodes in such a way that stimulation is initially performed with a low current of 10 mA, a pulse width of 250 µs and a frequency of 25 Hz. Then the intensity was increased until contractions of the stimulated muscles became visible (i.e., motor threshold + 20% amplitude). A stimulation maximum of 130 mA was not exceeded. To prevent painful stimulation in non-communicating patients, a special pain grading scale for coma patients was collected before and during stimulation (Nociception Coma Scale- Revised, NCS-R).

In the RTT-F group, concurrent to the verticalization a sham stimulation was applied with a minimum current intensity. This stimulation merely produced an electrifying sensation of the skin without being able to excite muscles (visual and palpatory control of the target muscles; settings: motor threshold -20% of amplitude).

In the control condition (CPT), patients received 45 min of physiotherapy according to standard clinic procedures. Physiotherapists in the CPT setting were given the aim to mobilize and verticalize patients as much as possible. Patients were mobilized to the edge of the bed or to a standing position using aids (e.g., splints and conventional standing devices). Verticalization was then defined as a position with the patient’s upper body being verticalized to more than 60° to the best upright position possible. The interventions took place in the morning in a time slot between 8 and 11 a.m. in all groups.

The study intervention was administered as part of a comprehensive early neurological rehabilitation program with an average of 300 min of therapy per inpatient day. Standard rehabilitation program consisted of occupational therapy, speech and language therapy, neuropsychological therapy, music therapy, and dysphagia therapy, in an individual composition depending on weekly rehabilitation goals. Patients of all three study groups received this rehabilitation program.

### Assessment of primary and secondary outcomes

After initial screening and inclusion into the study, baseline data (T0) were collected on the day of the first intervention, directly before treatment in a supine position. For patients in the RTT groups data were collected while the patient was already on the device but still in a horizontal position (< 30°) without FES. For the patients in the control group (CPT) data were collected in bed on the ward. The second time point of the study examination (T1) was during verticalization of the first intervention day. Patients were in a vertical (> 60°) upright position for 15–30 min before data was collected. To assess the direct effect of verticalization, data were collected subsequently after this first verticalization, again in a supine, horizontal position (< 30°; T2). The next day after the eighth day of the intervention, data were collected (T3) as well as after the sixteenth day, which was the day after the last session of the intervention (T4). Six months after study inclusion, follow-up data were collected to investigate long-term effects (T5). This last data collection took place at the patients' home.

#### Primary behavioral outcome

Change in the state of consciousness was defined as the primary outcome. The LoC was assessed by means of the CRS-R scale. The CRS-R [14] is the current gold standard in the assessment of DoC. It comprises six hierarchically structured subscales (auditory, visual, motor, verbal perception, communication, and arousal) to assess the level of consciousness in each of them. The highest level obtained in any of the subscales determines the current LoC. The CRS-R total score ranges from 0 to 23, with higher scores pointing towards increased LoC, however, it is not linear. A transformation of the CRS-R total score has been introduced recently and allows to express the total score on a linear basis from 0 to 100 [15]. CRS-R allows for clear distinction between the following states of consciousness: UWS, MCS, and emergence from MCS (eMCS). The latter is defined as regaining the ability for functional communication or functional object use.

For analysis, the proportion of patients who improved by at least one diagnostic category (according to the CRS-R) was determined, i.e., either from UWS at least to MCS or from MCS to eMCS. The CRS-R modified score was used to determine the LoC in greater detail. To quantify patients’ evolution during the intervention, the difference between the CRS-R assessment at T0 and T3 or T4 was determined by subtracting the CRS-R modified score at T0 from the one at T3 orT4, respectively.

#### Secondary behavioral outcomes

The Functional Independence Measure (FIM) was used to quantify independence in everyday life activities [16]. The Modified Ashworth Scale (MAS) [17] was used to assess the grade of spasticity. The degree of spasticity was rated for the left and right elbow and knee by passive flexion and extension. These four MAS scores of all extremities were added up to form a single score of spasticity. Additionally, the scores for the lower extremities were added up to measure the degree of spasticity of the legs only. T0 values were subtracted from T4 values to quantify patients’ change in spasticity during the interventional program.

We analyzed group differences in the change of blood pressure (RR) when patients were verticalized the first time. We computed mean blood pressure (systolic and diastolic) and heart rate (as beats per minute, bpm) before and after verticalization by taking the mean from all available treatment days. Similarly, mean RR and heart rate during verticalization were computed. This resulted in a mean systolic/diastolic RR value per patient before, during, and after verticalization. Furthermore, orthostatic hypotension due to the verticalization was defined as a 30 mmHg drop in systolic or 15 mmHg drop in diastolic RR, or an increase of 30 bpm in heartrate when the patient is verticalized, respectively [18].

Documentation further included any additional therapy type and quantity, and the duration in a position > 60° per day. The verticalization time > 60° in minutes was recorded within the ErigoPro® proprietary software in the RTT groups and with a watch (in minutes) in the CPT group. At follow-up (T5) it was noted, where the patient was living or whether the patient had died since the last study visit.

#### Secondary electrophysiological outcome

As a surrogate marker for changes of brain states on a subclinical electrophysiological level, quantitative high density electroencephalography data (HD-qEEG) were recorded at a sampling rate of 250 Hz with a 256-channel geodesic sensor net with Net Amps 400 amplifier and Net Station 4.5. Software (Electrical Geodesic Inc., Eugene, OR, USA). During recording, electrodes were referenced to the vertex and impedance was kept below 50 µV. HD-qEEG was recorded at T0 with the patient in supine position and verticalization < 30°, and at the end of the intervention period (T4) after the last verticalization session. HD-qEEG was recorded for 10 min at both timepoints.

To discover possible changes in the EEG signal, power spectrum and connectivity analyses were performed on EEG data of timepoints T0 and T4. The Mohawk pipeline was used to calculate the connectivity and power measures [19]. This is a toolbox implemented as a stand-alone application into MATLAB (MathWorks, version 2020a) using EEGLAB [20] and Fieldtrip [21] functions. For a detailed description of the HD-EEG preprocessing, refer to the original work [19]. Finally, relative amount of power dedicated to each frequency band was determined. Connectivity between pairs of channels was calculated for each band in all channels using the debiased weighted Phase Lag Index (dwPLI). The resulting connectivity matrix was collapsed to distinct frequency bands (i.e., delta, theta, alpha, beta, and gamma) and the median voltage was calculated for each frequency band. Differences in power and connectivity values between T0 and T4 were calculated to estimate the direct effect of verticalization.

### Statistical analyses

**Fig. 2:**
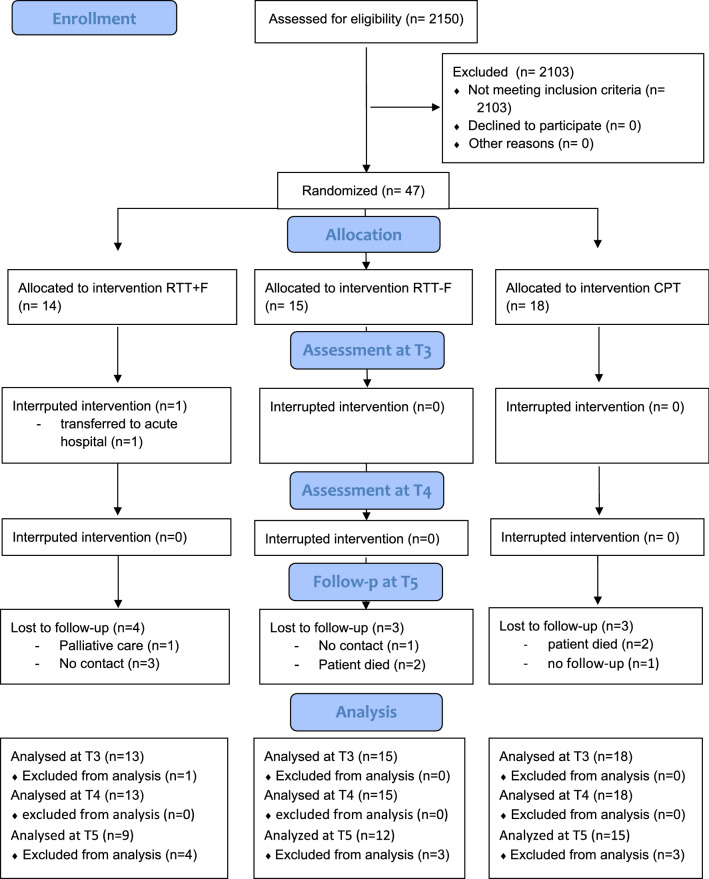
CONSORT-guidelines based flow of patients through the study. *RTT+F* Erigo®Pro treatment with functional electrical stimulation, *RTT-F* Erigo®Pro treatment without functional electrical stimulation, *CPT* conventional physiotherapy, *T3* time-point after 2 weeks of treatment, *T4* time-point of treatment finalization four weeks after inclusion, *T5* time-point after treatment follow-up

After data collection eleven patients had to be excluded from further analyses because of missing data (interruption during the study period due to transfer to the acute hospital at T3, n = 1; drop-out follow-up at T5, *n* = 10). For EEG analyses 12 patients had to be excluded due to missing data. See Fig. 2 for the patients’ flowchart. Finally, 46 patients who completed the treatment (assessments at T4) and 36 patients at follow-up (assessment at T5) were included into the intention-to-treat analyses, respectively.

Demographic, behavioral and outcome data were checked for normal distribution and homogeneity of variances. If normality or homogeneity of variances could not be assumed, non-parametric tests were applied (i.e., Kruskal–Wallis rank sum test when comparing three treatment groups and Wilcoxon rank sum and signed rank test when comparing the two RTT treatment groups against the control group). When testing for the improvement in DoC categories, a proportional test (Pearson’s Chi-squared Test for count data) was applied. Correlations between outcome variables (time of verticalization with CRS-R modified score, gamma band functional connectivity or spasticity) were tested using the Spearman correlation coefficient.

Since total randomization numbers were low, we also performed a secondary exploratory analysis, where both RTT groups (−F and +F) were combined and compared to the control group (CPT).

## Results

### Effect of RTT on levels of consciousness

Treatment groups were well balanced for clinical characteristics at baseline assessment apart from a longer time since injury in the RTT-F group and a significantly lower diastolic blood pressure in the CPT group (Table 1).

**Table 1:** Descriptive data of included patients randomly assigned to one of the three groups RTT+F, RTT-F, CPT

	RTT+F	RTT-F	CPT	All groups	*p* value^a^	Combined RTT+F RTT-F	*p* value^b^
N	14	15	18	47		29	
Age (years)	50 ± 14	47 ± 13	51 ± 18	49 ± 15	0.792	49 ± 13	0.654
Sex (female:male)	4:10	5:10	7:18	16:31	0.828	9:20	0.814
# TBI (%)	3 (21)	2 (13)	1 (6)	6 (13)	0.409	5 (17)	0.473
# Trachestomy tube (%)	12 (86)	12 (80)	17 (94)	41 (87)	0.455	24 (83)	0.473
TPI (days)	46 ± 32	177 ± 340	45 ± 16	87 ± 199	**0**.**021**	114 ± 250	0.533
UWS (%)	13 (93)	14 (93)	15 (83)	42 (89)	0.572	27 (93)	0.569
CRS-R MS (0–100)	3.2 ± 3.12	3.62 ± 3.06	6.68 ± 8.49	4.69 ± 5.9	0.290	3.44 ± 3.04	0.170
MAS (0–16)	1.3 ± 2.8	3.6 ± 5.0	1.3 ± 2.4	2.0 ± 3.6	0.569	2.5 ± 4.2	0.462
RR (mmHg)	120.68 ± 15.81/79.17 ± 7.93	123.42 ± 12.59/80.59 ± 11.22	117.71 ± 11.62/72.72 ± 8.21	120.41 ± 13.15/77.11 ± 9.71	0.284/**0.039**	122.15 ± 13.97/79.93 ± 9.68	0.163 /**0.011**
Heart rate (bpm)	90.90 ± 9.65	89.91 ± 6.39	89.60 ± 8.08	90.0 ± 7.90	0.674	90.37 ± 7.93	0.551

*RTT+F* Erigo®Pro treatment with functional electrical stimulation, *RTT-F* Erigo®Pro treatment without functional electrical stimulation, *CPT* conventional physiotherapy, *N* number of patients, *TBI* traumatic brain injury, *TPI* time post-injury, *UWS* unresponsive wakefulness syndrome, *CRS-R MS* Coma Recovery Scale-Revised Modified Score, *MAS* Modified Ashworth Scale, *RR* blood pressure (systolic/diastolic)

^a^*p* values for group comparison with all three treatment groups

^b^*p* values for group comparisons with the RTT groups combined. *p* values in bold denote the level of significance < 0.05

**Fig. 3:**
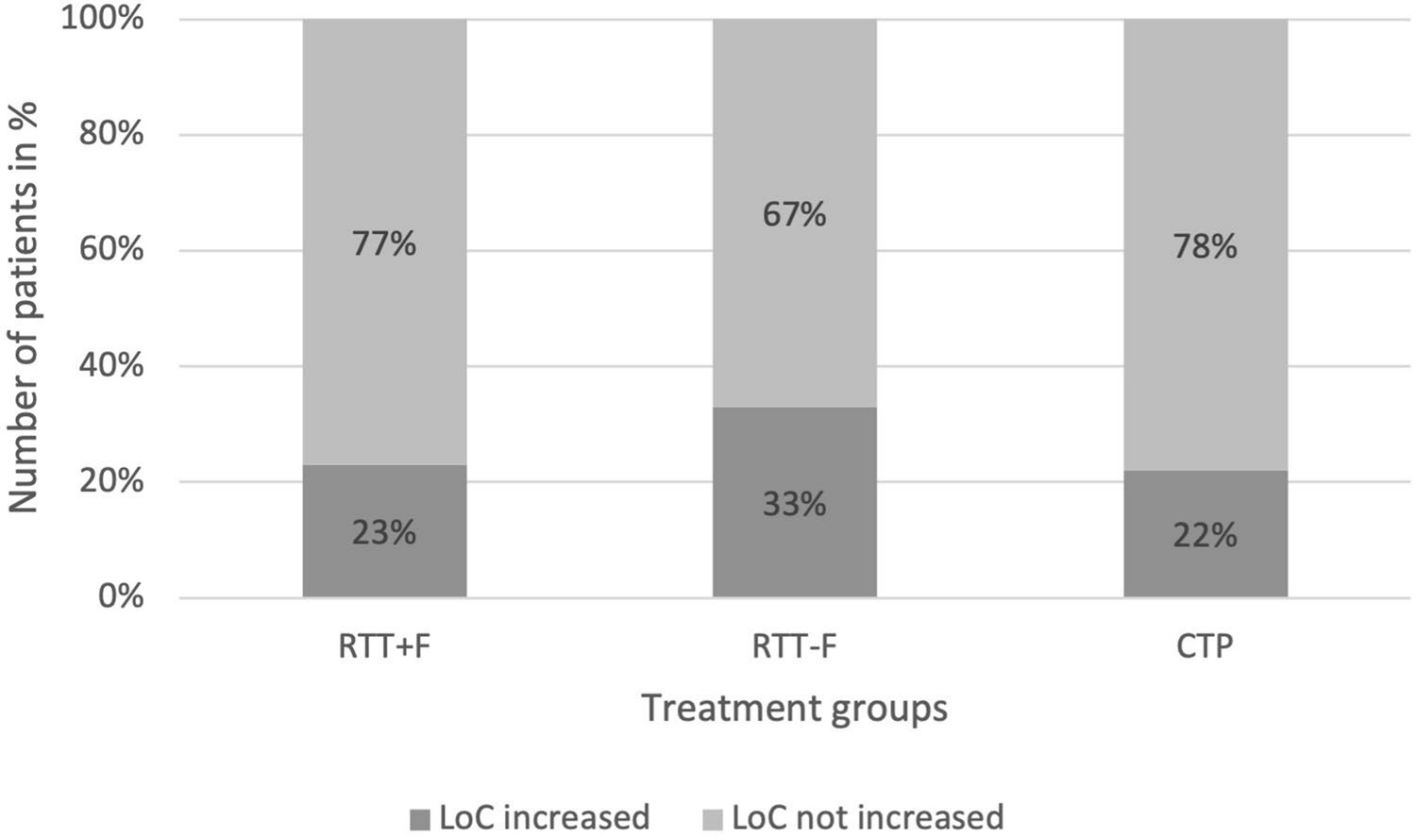
Number of patients for whom the level of consciousness (LoC) had (not) increased from baseline (T0) to end of treatment (T4) by at least one category as determined by the Coma Recovery Scale-Revised (CRS-R). Change of category can be unresponsive wakefulness (UWS) to minimally conscious state minus (MCS-), MCS- to minimally conscious state plus (MCS+), or MCS + to emergence from minimally conscious state (eMCS). *RTT+F* Erigo®Pro treatment with functional electrical stimulation, *RTT-F* Erigo®Pro treatment without functional electrical stimulation, *CPT* conventional physiotherapy

The number of patients who improved at least one category of consciousness from baseline to one day after the eighth intervention (T3) and from baseline to the day after the last intervention session (T4) did not differ between the three treatment groups (T3: *p* = 0.079, Fisher’s Exact Test; T4: *X*^2^(2) = 1.311, *p* = 0.519; Table 2). In all groups about one third of patients improved by at least one category of the CRS-R from baseline to end of treatment (see Fig. 3). Regarding the change from baseline to one day after the eighth session (T3) and from baseline to end of treatment (T4) in the CRS-R modified score (i.e., the continuous variable of the CRS-R), there was no significant difference between groups (T3: *X*^2^(2) = 0.123, *p* = 0.940; T4: *X*^2^(2) = 0.691, *p* = 0.708).

**Table 2:** Training and clinical outcome data of the three groups (RTT + F, RTT-F, CPT)

	RTT+F	RTT-F	CPT	*p* value^a^	Combined RTT+F/RTT-F	*p* value^b^
*N*	13	15	18		28	
Erigo®-training (min) (M ± SD)	363 ± 195	451 ± 173	–	0.430	405 ± 187	–
Erigo®-training (steps) (M ± SD)	6609 ± 4407	9523 ± 4271	–	0.094	8012 ± 4318	–
Verticalization > 60° (min) (M ± SD)	174 ± 108	229 ± 100	177 ± 90	0.969	203 ± 106	0.477
Treatment sessions (M ± SD)	9.64 ± 4.4	11.3 ± 3.9	14.9 ± 1.8	** < 0.001**	10.5 ± 4	** < 0.001**
CRS-R MS at T3 (M ± SD)	20.33 ± 33.69	8.34 ± 12.85	7.88 ± 10.42		13.88 ± 24.95	
CRS-R MS at T4 (M ± SD)	28.50 ± 41.08	18.18 ± 27.93	15.42 ± 21.34		22.91 ± 34.18	
CRS-R MS at T5 (M ± SD)	59.94 ± 44.63	36.1 ± 41.85	32.74 ± 41.23		45.63 ± 43.49	
DoC level improved (T0 to T3)	3/13	2/15	0/18	0.079	5/28	0.141
DoC level improved (T0–T4)	3/13	5/15	3/18	0.519	8/28	0.569
DoC level improved (T0–T5)	5/9	5/12	5/15	0.303	10/21	0.192
CRS-R MS (T3–T0) (M ± SD)	17.08 ± 33.93	4.71 ± 12.44	1.06 ± 2.55	0.940	10.42 ± 25.03	0.858
CRS-R MS (T4–T0) (M ± SD)	25.09 ± 41.13	14.54 ± 27.08	8.67 ± 18.48	0.708	19 ± 33.07	0.426
CRS-R MS (T5–T0) (M ± SD)	55.87 ± 44.51	32.42 ± 41.1	25.4 ± 37	0.325	26.53 ± 13.89	0.240
MAS (T4–T0) (M ± SD)	1.8 ± 4.2	0.3 ± 3.9	1.6 ± 3.9	0.053	0.9 ± 4.1	0.230
FIM (T4–T0) (M ± SD)	0 ± 0	0.2 ± 0.8	0.2 ± 0.7	.640	0.1 ± 0.6	.754
RR (mmHg; T1–T0) (M ± SD)	− 2.59 ± 10.07/1.14 ± 6.34	1.68 ± 5.99/4.07 ± 5.12	− 1.73 ± 7.24 /2.31 ± 6.39	185/0.245	− 0.30 ± 8.27 /2.71 ± 5.80	0.541/0.973
Heart rate (bpm; T1–T0) (M ± SD)	8.30 ± 6.04	7.31 ± 7.12	4.01 ± 5.36	0.134	7.77 ± 6.54	0.**046**

*RTT+F* Erigo®Pro treatment with functional electrical stimulation, *RTT-F* Erigo®Pro treatment without functional electrical stimulation, *CPT* conventional physiotherapy, *N* number of patients, *min* minutes, *DoC* disorders of consciousness, *MAS* Modified Ashworth Scale, *FIM* Functional Independence Measure, *RR* blood pressure (systolic/diastolic)

^a^*p* values for group comparison with all three treatment groups

^b^*p* values for group comparisons with the RTT groups combined. T0 = time-point of inclusion before start of treatment, T3 = time-point after two weeks of treatment, T4 = time-point of treatment finalization four weeks after inclusion, T5 = time-point after treatment follow-up. *p* values in bold denote the level of significance < 0.05 or < 0.001

When combining both RTT groups (with and without FES) into a single RTT group, there was no significant difference in improvement of LoC from T0 to T3 (*p* = 0.141, Fisher’s Exact Test) or from T0 to T4 (*X*^2^(1) = 0.255, *p* = 0.614), nor a change in CRS-R modified score from T0 to T3 (*W* = 241.5, *p* = 0.858) and T0 to T4 (W = 247.5, *p* = 0.426) compared to the CPT group.

The number of patients, who gained at least one LoC at long-term follow-up was not significantly different between treatment groups (RTT+F: 63%, RTT-F: 42%, CPT: 33%; *p* = 0.303). Similarly, the CRS-R modified score change from baseline (T0) to follow-up (T5) was not significantly different between groups (*X*^2^(2) = 2.247, *p* = 0.325). When combining both RTT groups (with and without FES) into a single RTT group, there was no significant difference in improvement of LoC (OR = 0.386, *p* = 0.192) nor a change in CRS-R modified score (W = 185.5, *p* = 0.240) compared to the CPT group.

When excluding two potential outliers with 477 and 1338 days post-injury, the results of all above-reported models remain unchanged regarding statistical significance.

### Effect of RTT on secondary outcomes

**Fig. 4:**
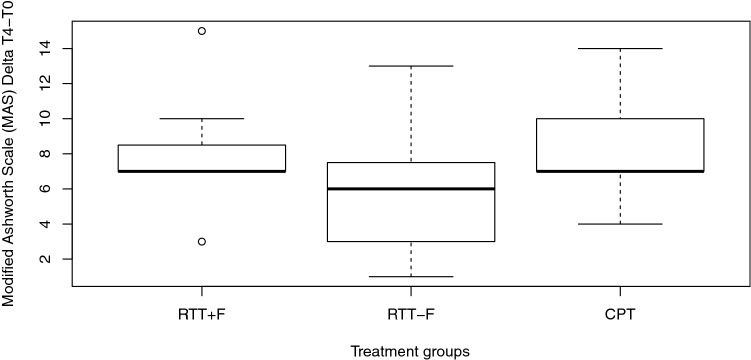
Change scores in spasticity as measured with the Modified Ashworth Scale (MAS) between baseline (T0) and end of treatment (T4). The rectangles represent the interquartile range; the bold horizontal lines inside the rectangles show the medians of each group, empty circles indicate potential outliers whereas the whiskers, which represent 1.5 distance from IQR1 and IQR3, are calculated by the default boxplot R function. *RTT+F* Erigo®Pro treatment with functional electrical stimulation, *RTT-F* Erigo®Pro treatment without functional electrical stimulation, *CPT* conventional physiotherapy

There was no significant treatment effect on any of the secondary outcomes (Table 2), except for a statistical trend in the group difference on MAS change scores (T4-T0; *X*^2^(2) = 5.891, *p* = 0.053; Fig. 4). The RTT+F and CPT groups showed an increase in spasticity compared to the RTT-F group. Apart from this observation, there was no significant treatment effect on any of the other long-term secondary outcomes.

### Effect of RTT on HD-qEEG measures for connectivity and spectral power

As the analyses were explorative in nature, p-values were not corrected for multiple comparisons. All three treatment groups did not differ significantly in their mean change of EEG power from pre to post treatment (all *p* > 0.05). However, the change rates on the connectivity values differed significantly between groups for gamma-band EEG connectivity (*X*^2^(2) = 8.323, *p* = 0.016). A post-hoc test revealed a significant difference in gamma-band connectivity between the RTT+F and the CPT group (*p* = 0.011, Bonferroni-corrected). Group differences in connectivity on the remaining frequency bands did not reach significance (all *p* > 0.05).

When comparing the combined RTT groups (RTT+F/RTT-F) to the control group there were no significant group differences in mean EEG power or connectivity for the distinct bands (all *p* > 0.05), except regarding connectivity in the gamma band (*W* = 76.5, *p* = 0.018). Details for the exploratory analyses can be found in Table 3.

**Table 3:** EEG outcome data of included patients randomly assigned to one of the three groups RTT+F, RTT-F, CPT

	RTT+F	RTT-F	CPT	*p* value	RTT+F/RTT-F	*p* value
Delta coherence (T4–T0)	− 0.043 ± 0.253	− 0.014 ± 0.062	0.018 ± 0.242	0.533	− 0.027 ± 0.176	0.377
Theta Coherence (T4–T0)	− 0.104 ± 0.268	0.017 ± 0.243	− 0.019 ± 0.390	0.735	− 0.041 ± 0.256	0.686
Alpha coherence (T4–T0)	− 0.021 ± 0.295	0.034 ± 0.210	− 0.106 ± 0.308	0.407	0.008 ± 0.249	0.222
Beta coherence (T4–T0)	− 0.032 ± 0.166	0.051 ± 0.193	− 0.016 ± 0.244	0.213	0.012 ± 0.181	1
Gamma coherence (T4–T0)	− 0.072 ± 0.109	0.003 ± 0.193	0.04 ± 0.075	**0.016**	− 0.033 ± 0.16	**0.018**
Delta power (T4–T0)	− 0.034 ± 0.051	− 0.011 ± 0.037	− 0.01 ± 0.048	0.613	− 0.02 ± 0.04	0.579
Theta power (T4–T0)	0.022 ± 0.033	0.0 07 ± 0.03	0.008 ± 0.037	0.496	0.01 ± 0.03	0.736
Alpha power (T4–T0)	0.01 ± 0.016	0.002 ± 0.007	0.001 ± 0.009	0.461	0.004 ± 0.01	.245
Beta power (T4–T0)	0.002 ± 0.002	0.0003 ± 0.004	0.0004 ± 0.004	.641	0.0 ± 0.0	0.598
Gamma power (T4–T0)	0.0002 ± 0.0006	0.004 ± 0.001	0.001 ± 0.003	0.573	0.0 ± 0.0	0.310

Values are given as mean with one standard deviation (M ± SD)

*RTT+F* Erigo®Pro treatment with functional electrical stimulation, *RTT-F* Erigo®Pro treatment without functional electrical stimulation, *CPT* conventional physiotherapy, *N* number of patients, *T0* time-point of inclusion before start of treatment, *T4* time-point of treatment finalization four weeks after inclusion. *p* values in bold denote the level of significance < 0.05

### Group differences regarding training characteristics

Regarding training characteristics, the number of therapeutic sessions was higher in the CPT group compared to the RTT groups (14.9 ± 1.8 vs. 10.5 ± 4; *p* < 0.001; see Table 2). All other training characteristics were similar between the study groups.

### Group differences regarding hemodynamic parameters

Between groups, there was no significantly different change in RR when verticalized for the first time (*p* > 0.05). Change in heart rate was significantly different between groups (*W* = 341, *p* = 0.046). The CPT group showed a trend towards lower increase in heart rate than the RTT+F group, (*p* = 0.059, Bonferroni-corrected; see Table 2). There was only one patient in the control group, whose blood pressure indicated an orthostatic hypotension continuously over the treatment period (RR 117/70 mmHg before to 97/55 mmHg during verticalization). Heart rate was stable in this patient (91 bpm to 94 bpm during verticalization).

### Correlation analysis between time of verticalization and improvement in levels of consciousness

**Fig. 5:**
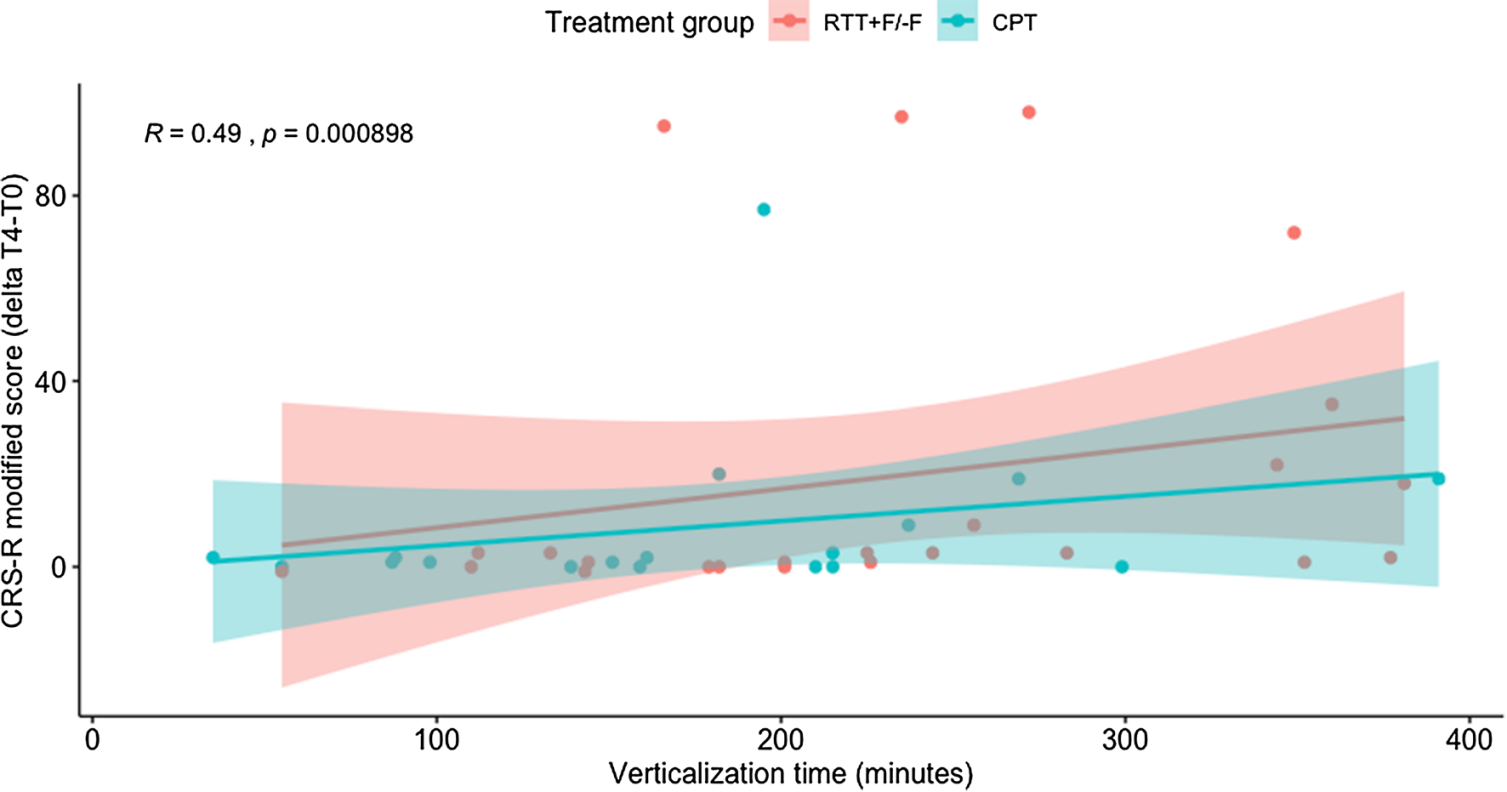
Correlation between verticalization time (minutes) during the four-week treatment period and the change in the Coma Recovery Scale-Revised (CRS-R) modified score between baseline and end of treatment. RTT+F/−F = robotic tilt table groups with (+F) and without (−F) functional electrical stimulation of the lower limbs. *CPT* conventional physiotherapy

Verticalization time across all treatment groups was significantly positively correlated with the increase in CRS-R modified scores from T0 to T4 (rho = 0.494, *S* = 6243.2, *p* < 0.001; Fig. 5). When looking at this effect on the level of treatment subgroups, there was a significant correlation in the RTT-F (rho = 0.639, *S* = 131.4, *p* = 0.009) and if both RTT groups were combined (rho = 0.536, *S* = 1066.7, *p* = 0.003). The correlations between recovery from T0-T4 and verticalization in the RTT+F and CPT treatment groups were not significant (all p-values > 0.05). There was a correlation trend between change in CRS-R modified scores and the amount of verticalization over 60° measured in minutes from T0 to T3 (rho = 0.208, S = 11,240, *p* = 0.088) as well as from T0 to T5 (rho = 0.247, *S* = 5378.2, *p* = 0.077). Time of verticalization was not significantly correlated with the change in gamma-band connectivity (rho = -0.058, *S* = 7553, *p* = 0.630). After excluding five potential outliers with very high rates of recovery from T0 to T4, the effect on the CRS-R modified score remained statistically unchanged (*p* < 0.001). Correlation analyses in the treatment subgroups were not affected after exclusion of these potential outliers (Table 4).

**Table 4:** Patient clinical information at admission

Patient	Age	Sex	Diagnosis	Etiology	TPI	DoC at T0	CRS-R at T0	CRS-R MS at T0	DoC at T4	CRS-R MS at T4	DoC at T5	CRS-R MS at T5
1	44	M	HIE	NT	43	UWS	3	1.71	UWS	1.71	UWS	1.71
2	66	F	Stroke	NT	93	UWS	1	1.04	UWS	4.17	MCS−	22.55
3	33	M	TBI	T	73	UWS	4	4.17	UWS	4.50	NA	NA
4	31	M	HIE	NT	27	UWS	1	0.33	UWS	3.80	UWS	4.84
5	69	M	Stroke	NT	33	UWS	3	3.13	MCS+	74.66	eMCS	83.00
6	85	M	Stroke	NT	45	UWS	6	5.54	eMCS	83.00	NA	NA
7	24	M	HIE	NT	32	UWS	2	1.37	UWS	4.84	eMCS	100.00
8	51	M	Stroke	NT	1338	UWS	6	4.84	MCS–	NA	MCS–	21.51
9	65	M	HIE	NT	38	UWS	4	3.46	UWS	3.80	UWS	4.17
10	30	F	HIE	NT	40	UWS	3	3.13	UWS	3.13	UWS	4.50
11	38	M	HIE	NT	46	UWS	6	4.84	UWS	4.84	UWS	2.75
12	62	M	Stroke	NT	165	UWS	4	2.75	UWS	4.50	UWS	4.84
13	50	F	HIE	NT	27	MCS–	8	21.88	MCS–	23.60	MCS–	22.55
14	58	M	TBI	T	30	UWS	4	4.17	UWS	5.54	UWS	5.54
15	48	M	TBI	T	31	UWS	2	2.08	eMCS	100.00	eMCS	100.00
16	72	M	HIE	NT	37	UWS	4	2.75	UWS	4.84	UWS	4.84
17	63	M	HIE	NT	38	UWS	0	0.00	MCS–	21.51	eMCS	100.00
18	56	M	TBI	T	21	MCS–	5	20.47	MCS–	29.51	eMCS	83.00
19	45	F	Stroke	NT	56	MCS–	7	13.18	MCS–	13.88	eMCS	74.99
20	62	F	Stroke	NT	43	UWS	4	3.46	MCS–	22.21	eMCS	48.94
21	40	F	HIE	NT	75	UWS	3	2.41	UWS	3.80	UWS	3.80
22	56	M	TBI	T	35	UWS	1	1.04	UWS	NA	NA	NA
23	47	M	HIE	NT	41	UWS	4	4.17	UWS	NA	NA	NA

Patient	Age	Sex	Diagnosis	Etiology	TPI	DoC at T0	CRS-R at T0	CRS-R MS at T0	DoC at T4	CRS-R MS at T4	DoC at T5	CRS-R MS at T5
24	69	M	HIE	NT	49	UWS	4	2.75	UWS	3.46	NA	NA
25	27	F	Stroke	NT	56	UWS	6	4.84	MCS–	22.55	eMCS	83.33
26	77	M	HIE	NT	42	UWS	1	1.04	UWS	3.46	NA	NA
27	27	M	HIE	NT	26	UWS	2	2.08	UWS	3.46	UWS	3.80
28	43	M	HIE	NT	49	UWS	4	3.46	UWS	3.80	UWS	3.80
29	25	F	Stroke	NT	48	UWS	7	5.88	UWS	5.54	eMCS	99.67
30	62	M	HIE	NT	28	UWS	6	4.50	eMCS	99.67	eMCS	99.67
31	65	F	Stroke	NT	35	UWS	1	1.04	UWS	0.00	NA	NA
32	35	M	Stroke	NT	41	MCS–	7	13.18	MCS–	48.61	eMCS	83.00
33	37	M	HIE	NT	37	UWS	3	3.13	UWS	3.80	UWS	3.80
34	54	F	TBI	T	477	UWS	2	1.37	UWS	4.84	UWS	4.84
35	46	M	Stroke	NT	84	UWS	4	2.75	UWS	1.37	NA	NA
36	34	F	HIE	NT	72	UWS	1	1.04	UWS	3.80	MCS–	14.22
37	31	F	Stroke	NT	148	UWS	4	2.75	UWS	NA	UWS	NA
38	42	F	Stroke	NT	88	MCS–	11	30.89	UWS	49.65	eMCS	100.00
39	47	M	HIE	NT	49	UWS	5	3.80	MCS–	23.60	eMCS	100.00
40	49	M	HIE	NT	33	UWS	1	1.04	UWS	4.50	NA	NA
41	69	M	HIE	NT	45	UWS	4	3.46	UWS	4.84	NA	NA
42	49	M	Stroke	NT	32	UWS	3	3.13	UWS	3.13	UWS	2.08
43	57	F	Stroke	NT	46	UWS	4	3.46	eMCS	100.00	eMCS	100.00
44	47	M	Stroke	NT	39	UWS	6	4.84	MCS–	14.22	UWS	4.84
45	50	F	Stroke	NT	85	UWS	3	2.41	UWS	NA	NA	NA
46	32	M	HIE	NT	41	UWS	2	1.37	UWS	1.71	UWS	2.75
47	49	F	HIE	NT	50	UWS	5	3.80	UWS	3.46	UWS	4.50

*DoC* Disorder of Consciousness, *CRS-R* Coma Recovery Scale-Revised, *CRS-R MS* Coma Recovery Scale-Revised Modified Score, *T0* time-point before first verticalization, *T4* time-point after end of treatment, *T5* time-point at 5 months follow-up after treatment, *M* male sex, *F* female sex, *HIE* hypoxic-ischemic encephalopathy, *TBI* traumatic brain injury, *T* traumatic brain injury, *NT* non-traumatic brain injury, *UWS* unresponsive wakefulness syndrome, *MCS−* minimally conscious state minus (lower end of the spectrum in minimally conscious state)

### Efficiency of verticalization

The efficiency of verticalization (tested as the total minutes of verticalization divided by the number of sessions) revealed a significant difference between the RTT and CPT groups (*F*(1,45) = 9.372, *p* = 0.004). Post-hoc pairwise t-tests revealed a significant difference in efficiency of verticalization between the RTT+F and CPT group (RTT+F = 17.2 ± 5.2 min, CPT = 11.8 ± 5.4; *p* = 0.008) and the RTT-F and CPT group (RTT-F = 20.0 ± 2.9, CPT = 11.8 ± 5.4; *p* < 0.001). The efficiency of verticalization in both RTT+F and RTT-F groups together were significantly higher than in the CPT group (RTT = 18.7 ± 4.4, CPT = 11.8 ± 5.4; *W* = 447, *p* < 0.001).

## Discussion

To the authors’ knowledge, this is the first randomized-controlled trial among patients with DoC using a RTT with built-in FES of the lower extremities. The primary goal of this study was to test whether RTT treatment was associated with improved recovery of consciousness compared to CPT as part of a comprehensive neurorehabilitation program. RTT with or without FES was equally effective as CPT in promoting recovery of consciousness. We found indirect evidence, that time spent in an upright body position was significantly correlated with the extent of recovery of consciousness at end of treatment and marginally significantly at follow-up. These results could be a hint for a beneficial effect of daily verticalization over 60° on LoC, pertaining up to 5 months after treatment. The significant correlation between recovery until the end of treatment and verticalization across groups could be a result of the strong and highly significant correlation within the RTT-F subgroup. This could be explained by longer verticalization times above 60° position in the RTT-F compared to the RTT+F and CPT groups, that may have been caused by longer preparation times in the RTT+F (due to placement of FES electrodes) and CPT (due to stabilization at bedside through therapists) subgroups, and consequently less verticalization time. We tested whether RTT+F and CPT treatment were less effective by comparing the mean verticalization times per session across groups. The RTT groups had comparable verticalization times, which were significantly longer than the CPT groups. The efficiency of the RTT treatment with regard to net therapy time is in line with previous research [22], and scientifically demonstrates its feasibility in the treatment of DoC patients. Likewise, the traditional therapeutic experience is that head-up-tilt seems to be associated with recovery of consciousness [4]. However, as there was no control group without any mobilization, it is hard to say if the observed rate of recovery is related to the intervention or if it should be interpreted as a sign of natural recovery. A conclusion in the sense of superiority of verticalization programs over a ‘no-therapy’ program cannot be drawn based on results of this RCT. A potential bias could have been caused by five cases with extreme evolutions of recovery from T0 to T4. This was checked by running the correlation analysis on a subsample without these sensitive cases. The positive correlation between time of verticalization over 60° and recovery remained statistically significant. Thus, the cases with extremely high rates of recovery most likely did not influence the relation between amount of verticalization and recovery.

A recent meta-analysis of different verticalization treatments in patients with DoC found only partial evidence for the effectiveness of verticalization treatment regimes [13]. The number of sessions in the reported studies varied from 15 to 24 sessions, with two of them showing a small [22] or large [23] effect size, respectively. In our study, however, the number of sessions was much lower, with a mean of 10–15 sessions, depending on the treatment group. The number of sessions was highest in the control group and lowest in the RTT+F group. The difference in amount of treatment sessions could explain why there was no superiority in the RTT training groups regarding recovery against the control group. It must be considered that the number of sessions could have had an influence on the rate of recovery, in the sense that less treatment sessions might have led to less recovery. Patients with less treatment sessions naturally exhibit less verticalization time. Therefore, it seems logical that less treatment sessions go along with less recovery, and thus is part of the positive correlation between amount of verticalization and recovery.

Still the question arises, whether the reported rate of recovery was clinically meaningful. Ng and King [13] claim that none of the reported studies in their systematic meta-analysis had a control group, so it could not be determined whether the rate of recovery promoted by a head-up tilt treatment was clinically meaningful or just an effect of spontaneous recovery over time. A prospective observational study in several neurorehabilitation centers in Germany showed that for 26% of recruited patients with severe acquired brain injury LoC improved for at least one category of the CRS-R within 6 months follow-up [24]. Recovery rates within the treatment period (4 weeks) were between 22 and 33% in this study, and are thus comparable with rates of recovery if patients only receive standard care [24]. Consequently, the rate of recovery reported in this study can be interpreted as the amount of spontaneous recovery that can be expected in a rehabilitation cohort of patients with prolonged DoC.

Recovery on secondary clinical outcomes (gain in functional independence during the treatment or change in the degree of spasticity of upper and lower limbs) was not better in the RTT groups compared to the CPT group. The RTT+F and the CPT group showed overall higher changes than the RTT-F (see Table 2), but the level of spasticity in the RTT-F group was already lower at T0 (not significant) than in the other groups (see Table 1). A recent study using a robotic training device together with RTT showed a decline in spasticity as well as a gain in functional independence [25]. It must be pointed out that in this study the amount of training was four times longer than the protocol used in our study, which could explain the positive effects on functional independence and spasticity. In a prospective observational study carried out on 102 DoC patients in (neuro)intensive care units across Italy, at discharge there was no significant difference in functional independence between a group with and without mobilization [11]. Notably, the mean total scores were at the lower end of the scale with 21 in the mobilization and 18.5 points in the no-mobilization group, which is comparable to our sample. This demonstrates the severity of injuries in our study population: at admission, all patients scored 18 points on the FIM assessment. Future studies could focus on patients who have already gained a higher state of recovery at the beginning of a verticalization program. There was no interruption of treatment in the RTT groups due to orthostatic hypotension, except one case in the control group, in line with literature reporting a lower amount of treatment interruptions, which is associated with fewer interruptions due to orthostatic hypotension [13]. Still, the increase in heart rate during verticalization was considerably higher in the RTT+F group than in the CPT group. This could be associated with the RTT cycling leg movement to which patients might have reacted with a higher heart rate than patients receiving CPT. This needs to be investigated in more detail in future clinical trials.

We did not find an effect on EEG beta band power, after RTT treatment, as did a study on the effect of the verticalization on beta power in the injured hemisphere of MCS patients [12]. For brain connectivity, we found a meaningful difference in the change of gamma-band coherence (30–45 Hz). Post-hoc comparisons revealed a significant difference between the RTT+F and the CPT group (change scores were highest for the control group, and lowest for the RTTF group). This could have been caused by significantly more sessions in the control group compared to the RTT groups (10.5 vs. 14.9 on average).

The biggest limitation of this study is the absence of a treatment arm without any kind of verticalization or mobilization except of standard therapy. Earlier work reported positive evidence for RTT treatments [23, 26]. Due to our study design with lack of a true control group, i.e., a group without efforts to verticalize patients, we could not directly determine the effect of verticalization as a treatment principle. Integrating a control group without any verticalization or mobilization could have demonstrated the benefit of verticalization in causal and not only correlative manner. Yet, integrating a control group without any kind of mobilization is ethically highly questionable for the following reasons. The first weeks and months after an acquired brain injury are extremely valuable for functional recovery. Verticalization during early rehabilitation leads to cardiovascular stability, helps regaining normal postures [11], and can also avoid contractions in the ankle joints [9] or pressure ulcers [8]. Thus, we decided not to include a control group without mobilization and the associated benefit.

Further, as pointed out above, the number of treatment sessions was greatly imbalanced, thus limiting the conclusions that can be drawn from this study. A possible explanation of the RTT groups having significantly less sessions than the control group could be the higher amount of protocol deviations in the RTT groups than in the control group.

Similarly, the time post-injury (TPI) was not well-balanced between groups, with the longest interval in the RTT-F group, whereas the TPI was quite similar in the RTT+F and CPT groups. At closer inspection, this imbalance is most likely caused by two potential outliers in the RTT-F group with very long TPI. Still, it is unlikely that these two outliers might have influenced the results in a negative way, as one of them had improved in LoC at T4 and one had not.

Another limitation of this study is the lack of blinding in outcome assessors. Blinding of researchers was not feasible during verticalization sessions (T1), however, for outcome assessments (CRS-R, NCS-R, MAS, FIM and EEG analysis) it would have been beneficial to have blinded assessors. A meta-analysis reported that in 8/10 articles there was no blinding of outcome assessors [13]. This should be improved in future research to avoid biased estimates of treatment effects.

In this study the most frequent reason for an interruption or premature stop of the treatment protocol was transferring patients back to acute-setting hospitals (e.g., for ventriculo-peritoneal shunting or sepsis). Minor deviations from the protocol occurred more often in the RTT groups than in the CPT group and could have limited the effectiveness of the Erigo®Pro-based treatment in improving consciousness. This must be investigated in more detail in future studies.

## Conclusion

Four weeks of RTT treatment to achieve head-up verticalization were not more effective in promoting recovery of consciousness in DoC patients than CPT. Correlation analysis implies that total therapy time with head-up tilt—irrespective of the method used—is associated with improved LoC. CPT seemed more feasible to achieve head-up tilt than RTT, as there were no interruptions during the intervention and the highest number of verticalization therapy sessions in the CPT group. However, the net therapy time that could be used to verticalize the patient was significantly longer in the RTT groups compared to CPT. Taken together, this small exploratory RCT does not support the hypothesis, that RTT therapy is necessary to promote recovery of consciousness. Though this was not a limiting factor in our study, RTT-based verticalization may be a method to provide therapy to DoC patients with severe hemodynamic orthostatic problems with high efficiency due to longer daily verticalization times compared to standard treatment.

## Data Availability

The datasets generated during and/or analyzed during the current study are available from the corresponding author on reasonable request.
